# Yacon (*Smallanthus sonchifolius*) Leaf Extract Attenuates Hyperglycemia and Skeletal Muscle Oxidative Stress and Inflammation in Diabetic Rats

**DOI:** 10.1155/2017/6418048

**Published:** 2017-07-20

**Authors:** Klinsmann Carolo dos Santos, Bianca Guerra Bueno, Luana Ferreira Pereira, Fabiane Valentini Francisqueti, Mariana Gobbo Braz, Lahis Fernandes Bincoleto, Lilian Xavier da Silva, Ana Lúcia A. Ferreira, Ana Cláudia de Melo Stevanato Nakamune, C.-Y. Oliver Chen, Jeffrey B. Blumberg, Camila Renata Corrêa

**Affiliations:** ^1^São Paulo State University (UNESP), Medical School, Botucatu, SP, Brazil; ^2^Antioxidants Research Laboratory, Jean Mayer USDA Human Nutrition Research Center on Aging, Tufts University, Boston, MA, USA; ^3^São Paulo State University (UNESP), Dentistry School, Araçatuba, SP, Brazil

## Abstract

The effects of hydroethanolic extract of Yacon leaves (HEYL) on antioxidant, glycemic, and inflammatory biomarkers were tested in diabetic rats. Outcome parameters included glucose, insulin, interleukin-6 (IL-6), and hydrophilic antioxidant capacity (HAC) in serum and IL-6, HAC, malondialdehyde (MDA), superoxide dismutase (SOD), catalase (CAT), and glutathione peroxidase (GPx) in soleus. The rats (10/group) were divided as follows: C, controls; C + Y, HEYL treated; DM, diabetic controls; and DM + Y, diabetic rats treated with HEYL. Diabetes mellitus was induced by administration of streptozotocin. C + Y and DM + Y groups received 100 mg/kg HEYL daily via gavage for 30 d. Hyperglycemia was improved in the DM + Y versus DM group. Insulin was reduced in DM versus C group. DM rats had higher IL-6 and MDA and lower HAC in the soleus muscle. HEYL treatment decreased IL-6 and MDA and increased HAC in DM rats. DM + Y rats had the highest CAT activity versus the other groups; GPx was higher in C + Y and DM + Y versus their respective controls. The apparent benefit of HEYL may be mediated via improving glucoregulation and ameliorating oxidative stress and inflammation, particularly in diabetic rats.

## 1. Introduction

Type 1 diabetes mellitus (T1DM) occurs by autoimmune-mediated destruction of pancreatic *β*-cells, leading to insulin deficiency and loss of glycemic control. The hyperglycemia that occurs in diabetes increases the production of reactive oxygen species (ROS) and weakens antioxidant defense, resulting in enhanced oxidative stress [1]. ROS mediate several biochemical and molecular pathways that can exacerbate oxidative stress [2], such as activating the transcription factor nuclear factor kappa B (NF-*κ*B), which increases the transcription of inflammatory cytokines and chemokines [3] promoting inflammation. Moreover, uncontrolled ROS generation could also attack the cellular proteins, lipids, and nucleic acids leading to cellular dysfunction including loss of energy metabolism, alteration on cell signaling and cell cycle control, mutations, and inflammation. In addition, it plays a role in several pathological processes in skeletal muscle [4]. These reactive species are important signaling molecules necessary for muscle function and for adaptive response to stress [5]. However, overproduction of ROS and decrease of the antioxidant defense have negative impact on muscle function, as impaired muscle growth and strength and altered metabolic capacity [6].

Medicinal plants are widely used as alternative therapeutics for the prevention or treatment of diseases. Recently, great attention has been paid to the use of natural compounds, due to their nutritional and pharmacological characteristics [7].

Yacon (*Smallanthus sonchifolius* [Poepp. & Endl.] H. Robinson, Asteraceae) is a native Andean plant cultivated for its tubers, which are commonly used as a food in South America. Some studies have reported the presence of large amounts of phenolic compounds in extracts from Yacon leaves and tubers, mainly chlorogenic, protocatechuic, ferulic, rosmarinic, gallic, gentisic, and caffeic acids and their derivatives [8]. Evidence has also emerged about the antioxidant activity [9], protective effects on oxidative damage and glucose metabolism in rat hepatocytes, and insulin-like effects of Yacon leaf extracts [10, 11].

Antioxidant compounds have long been known to diminish inflammatory and oxidative stress responses. In addition, antioxidants scavenge ROS and increase the capacity of the antioxidant defense enzyme system [2]. Therefore, antioxidants can help diminish oxidative damage and inflammation and slow or prevent the progression of diabetic complications.

Natural products are the groundwork of preventing and curing several diseases. Moreover, ethnopharmacological knowledge is one attractive way to enhance the probability of success in new drug-finding efforts. Regarding diabetic complications, plants that can be effectively used based on their therapeutic applications, for example, for diabetes, are worthy of special attention, and studies are needed regarding their side effects, the ability to maintain normal levels of glycemia, and their possible control on oxidative stress and inflammation. Considering the complications of type 1 diabetes and previous data on Yacon's activities, the present study was undertaken to elucidate the antioxidant, anti-inflammatory, and antihyperglycemic activity of hydroethanolic extract from* S. sonchifolius* leaves (HEYL) in the serum and skeletal muscle of STZ-induced diabetic rats.

## 2. Materials and Methods

### 2.1. Plant Material and Extract Preparation

The leaves of* S. sonchifolius* were collected in June (2014) by Klinsmann Carolo dos Santos in Curitiba, PR, Brazil. The specimen was provided by Dr. Átila Francisco Mógor from the Department of Plant Science and Crop Protection, Federal University of Paraná, Curitiba, Paraná, Brazil, and identified by Dr. Lin Chau Ming from São Paulo State University (UNESP), School of Agriculture, Botucatu, SP, Brazil, and the voucher specimen was deposited to the Herbarium at the São Paulo State University (UNESP), Institute of Biosciences, Botucatu, SP, Brazil, under the register 32752, for future reference. The leaves from* S. sonchifolius* were dried for seven days at 50°C, powdered (3 *µ*m), and subjected to percolation at room temperature using a mixture of ethanol : H_2_O (7 : 3, v/v) with a flux of 2.0 mL/min/kg. The solvents were evaporated to dryness under a low pressure (45°C) using rotary evaporator in vacuum system to afford the crude HEYL.

### 2.2. Characterization of Phenolics

For the characterization of phenolics, 10 mg HEYL was reconstituted in 1 mL methanol, followed by acidic hydrolysis with 1 mL of 2.4 M HCl at 80°C for 2 h in the dark. After the incubation, the solution was filtered through a 0.45 *µ*m nylon membrane (Millipore Corp., Bedford, MA) and then injected onto a HPLC system equipped with a Zorbax SB-C18 column (4.6 × 250 mm, 3.5 *µ*m) and a CoulArray 5600A electrochemical detector (ESA Inc., Chelmsford, MA). Phenolic acids and flavonoids were quantified according to the method of Li et al. (2009) [12]. The limits of quantitation for phenolic acid and flavonoids were 1 ng on column. The linearity of calibration curves of authentic standards with concentrations ranging from 0.01 to 2 ng/ml was at least ≥0.991. The identification of each compound was based on a comparison of the retention time and electrochemical response of the authenticated standards. The results are expressed in *µ*g/100 mg HEYL.

### 2.3. Dose-Response Profile of HEYL Treatment

To establish a dose-response profile for the antihyperglycemic activity of Yacon leaves, we used varying doses of HEYL (25, 50, and 100 mg/kg body weight/day constituted in 1 mL of 0.9% saline) to identify the lowest dose that could elicit an optimal antihyperglycemic effect. Fifteen male Wistar rats, 60 d of age, were maintained in an environmentally controlled room (22 ± 3°C; 12-hour light/dark cycle and relative humidity of 60 ± 5%) and were fed with a standard rat pellet diet (Purina Ltd., Campinas, SP, Brazil) and water ad libitum. The animals were randomly assigned to one of three groups: HEYL 25; HEYL 50; and HEYL 100.* Diabetes mellitus* was induced by one i.p. administration of streptozotocin (STZ; 40 mg/body weight), and the animals received HEYL for gavage for 2 weeks after the establishment of diabetic condition. We found that after 2 weeks of the treatment, the highest dose showed the most potent hyperglycemic effect in STZ model of diabetes. Thus, the subsequent experiments with HEYL were carried out with the dose of 100 mg/kg administered orally.

### 2.4. Animals and Experimental Groups

Forty male Wistar rats, 60 d of age, were maintained in an environmentally controlled room (22 ± 3°C; 12-hour light/dark cycle and relative humidity of 60 ± 5%) and were fed with a standard rat pellet diet (Purina Ltd., Campinas, SP, Brazil) and water ad libitum. The experimental protocol was approved by the Ethics Committee on the Use of Animals (CEUA) at the Botucatu Medical School, São Paulo State University (UNESP) under number 1082-2014 (approved in April 24, 2014). The animals were randomly assigned to one of four groups (*n* = 10): C (control group): normal rats; C + Y: normal rats receiving HEYL; DM: diabetic rats; and DM+Y: diabetics rats receiving HEYL.* Diabetes mellitus* was induced by i.p. administration of streptozotocin for one time (STZ; 40 mg/body weight). Blood glucose was measured 48 h and 7 days after the STZ administration. The animals with blood glucose greater than 250 mg/dL were considered diabetic. The animals received HEYL (100 mg/kg body weight/day constituted in 1 mL of 0.9% saline) for gavage for 30 days after the 7th day of established diabetic condition. Control animals were given the same volume of saline. The animals were fasted overnight and killed by decapitation after anaesthesia with ketamine (50 mg/kg) and xylazine (0.5 mg/kg) by intraperitoneal injection, and all efforts were made to minimize suffering. Blood was collected in tubes and then centrifuged at 3500 rpm ×g. The serum and soleus muscle were collected and stored at −80°C until analysis.

### 2.5. Preparation of the Soleus Muscle for Analysis

Soleus muscle was weighed (100 mg) and homogenized in 1.0 mL cold PBS (pH 7.4) using ULTRA-TURRAX® T25 basic IKA® Werke Staufen/Germany. After centrifugation at 800 ×g at 4°C for 10 min, the supernatant was collected for malondialdehyde (MDA) and IL-6 determinations. For the antioxidant enzymes determination, 100 mg soleus muscle was homogenized (1 : 10 v/v) in KH_2_PO_4_ (10 mmol/L)/KCl (120 mmol/L), pH 7.4, and centrifuged at 2.000 ×g for 20 min.

### 2.6. Biochemical Measurements in Serum and Soleus Muscle

An enzymatic colorimetric kit was used to measure serum glucose (Bioclin®, Belo Horizonte, Minas Gerais, Brazil). Insulin (Immuno-Biological Laboratories, Inc.) and IL-6 (R&D Systems, Inc.) were measured by an immunoassay, using a microplate reader (Spectra Max 190; Molecular Devices).

### 2.7. Pancreatic Beta-Cell Function

Pancreatic beta-cell function was determined using the index of homeostasis model assessment (HOMA) [13] using the following formula: HOMA-BETA (*Homeostasis Model Assessment* Beta-*Cell Function*) = 20 × Fasting Insulin (*µ*U/mL)/Fasting Glucose (mM) − 3,5.

### 2.8. Malondialdehyde (MDA) Analysis in Soleus Muscle

A 100 *μ*L aliquot of soleus muscle homogenate was used for MDA analysis. Briefly, we added 700 *μ*L of 1% orthophosphoric acid and 200 *μ*L of thiobarbituric acid (42 mM) to the sample and then boiled it for 60 min in a water bath; the sample was cooled on ice immediately after that. Two hundred *μ*L was transferred to a 2 mL tube containing 200 *μ*L sodium hydroxide-methanol (1 : 12 v/v). The sample was vortex-mixed for 10 s and centrifuged for 3 min at 13,000 ×g. The supernatant (200 *μ*L) was transferred to a 300 *μ*L glass vial and 50 *μ*L injected onto the column. The HPLC was a Shimadzu LC-10AD system (Kyoto, Japan) equipped with a C18 Luna column (5 *μ*m, 150 × 4.60 mm, Phenomenex Inc., Torrance, CA, USA), a Shimadzu RF-535 fluorescence detector (excitation: 525 nm, emission 551 nm), and 0.5 mL/min flow of phosphate buffer (KH_2_PO_4_ 1 mM, pH 6.8) [14]. MDA was quantified by area determination of the peaks in the chromatograms relative to a standard curve of known concentrations.

### 2.9. Measurement the Hydrophilic Antioxidant Capacity (HAC) in Serum and Soleus Muscle

The hydrophilic antioxidant capacity was determined fluorometrically, as described by Beretta et al. (2006) [15] using a VICTOR X2 reader (Perkin Elmer, Boston, MA). The antioxidant activity was quantitated by comparing the area under the curve relating to the oxidation kinetics of the suspension phosphatidylcholine (PC), which was used as reference biological matrix. The peroxyl radical 2′,2′-azobis-(2-amidinopropane) dihydrochloride (AAPH) was used as an initiator of the reaction. The results represent the percent inhibition (4,4 difluoro-5-(4-phenyl 1-3 butadiene)-4-bora-3,4-diaza-s-indacene) (BODIPY) 581/591 plasma with respect to the control sample of BODIPY 581/591 PC liposome. All analyses were performed in triplicate. The results are reported as percentage of protection.

### 2.10. Antioxidant Enzymes Activity Evaluation in Soleus Muscle

Superoxide dismutase activity was measured based on the inhibition of a superoxide radical reaction with pyrogallol and the absorbance values were measured at 420 nm [16]. The values are expressed as units per milligram of protein. Catalase activity was evaluated by following the decrease in the levels of hydrogen peroxide. The absorbance values were measured at 240 nm [17]. The activity is expressed as pmole of H_2_O_2_ reduced/min/mg protein. Glutathione peroxidase activity was measured by following *β*-nicotinamide adenine dinucleotide phosphate (NADPH) oxidation at 340 nm as described by Flohé and Günzler (1984) [18]; the results were expressed as *μ*mol hydroperoxide reduced/min/mg protein. Protein was quantified based on Lowry's method [19], using bovine serum albumin as the standard.

### 2.11. Statistical Analysis

Results are expressed as mean and standard error of the mean (SEM), and significance was calculated by two-way ANOVA followed by Holm-Sidak method. The software used was SigmaStat version 3.5 for Windows (Systat Software, Inc., San Jose, CA, USA). Differences were considered significant at *P* < 0.05.

## 3. Results

### 3.1. Glycemia, Insulin, and HOMA-BETA

**Figure 1:**
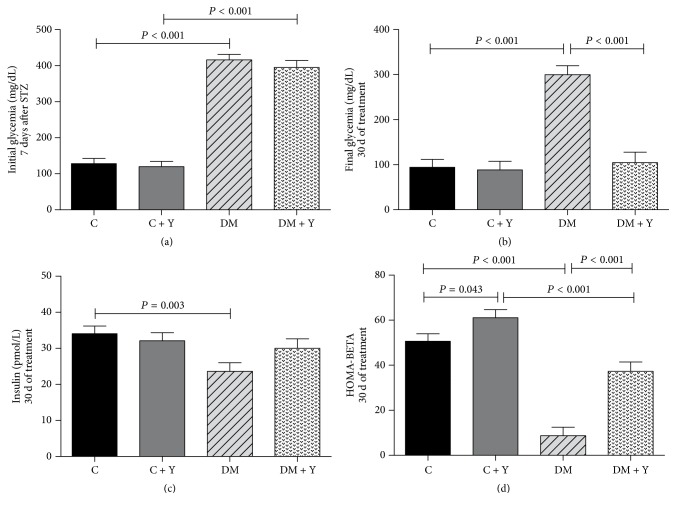
(a) Initial glycemia (7 d after STZ administration); (b) final glycemia (30 d of treatment); (c) insulin (30 d of treatment); (d) HOMA-BETA (30 d of treatment) of the different experimental groups. C (control group): normal rats; C + Y: normal rats receiving HEYL; DM: diabetic rats; and DM + Y: diabetics rats receiving HEYL. The results are expressed as the mean ± SEM.

STZ-induced diabetic rats (DM and DM + Y) showed 3.25- and 3.08-fold, respectively, higher blood glucose levels than the control groups (Figure 1(a)) in the beginning of the experiment (7 d after administration of STZ). After treatment with HEYL, the DM + Y animals showed reduction of glycemia to values similar to the controls (Figure 1(b)). The insulin was lower in DM when compared with the control groups (Figure 1(c)). The same was found for HOMA-BETA; DM presented the lowest values when compared to C and DM + Y, whereas the treated DM group presented increase of HOMA-BETA (Figure 1(d)).

### 3.2. Characterization of Phenolics

Ten phenolics in the hydroethanolic extract of Yacon were quantified using a HPLC-ECD method. The active principles with their concentrations, retention time (RT), and peak area (*µ*C) are presented in Table 1.

### 3.3. Antioxidant Enzymes and Lipoperoxidation Marker in Soleus Muscle

The treatment with Yacon leaves in DM + Y decreased MDA (Figure 3(d)) in soleus muscle when compared to DM, which presented the highest values for this variable. DM + Y group presented the highest catalase activity among the groups (Figure 3(b)), whereas the GPx (Figure 3(a)) was higher in C + Y and DM + Y. No significant difference was found for SOD (Figure 3(c)) among the groups.

### 3.4. Hydrophilic Antioxidant Capacity (HAC) and IL-6 in Serum and Soleus Muscle

**Figure 2:**
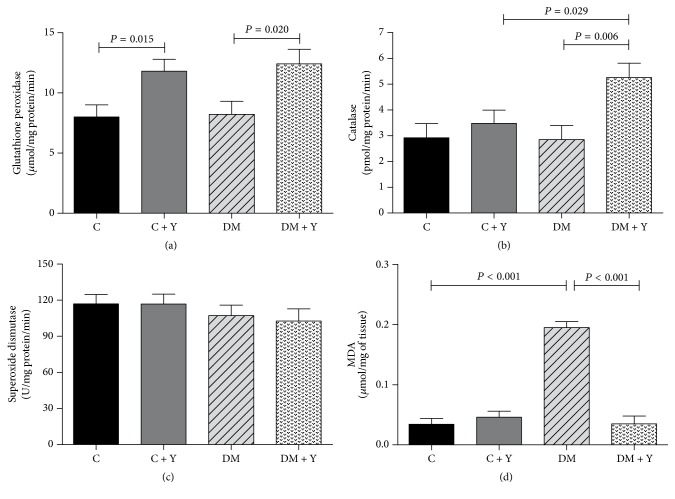
(a) Catalase (CAT) activity; (b) superoxide dismutase (SOD) activity; (c) glutathione peroxidase (GPx) activity; (d) MDA concentration in soleus of the different experimental groups. C (control group): normal rats; C + Y: normal rats receiving HEYL; DM: diabetic rats; and DM + Y: diabetics rats receiving HEYL. The results are expressed as the mean ± SEM.

There were no significant differences for plasma IL-6 (Figure 2(c)). Plasma HAC was higher in C + Y when compared to C group, although, when evaluated in soleus muscle, a 2.2-fold increase of IL-6, with a 29.1% decrease of HAC, was observed in DM group compared to C group (Figure 2). HEYL promoted decrease of IL-6 and increase of HAC in DM + Y group when compared to untreated group.

## 4. Discussion

Streptozotocin (STZ) is a widely used chemical for the induction of experimental diabetes [20]. Type 1 diabetes can be induced in rodents by a single STZ injection [21]. All these STZ-induced diabetic animal models have been useful in elucidating the mechanisms of diabetic pathogenesis and in screening natural products and pharmacological agents that are potentially capable of lowering blood glucose levels [22] and attenuating the oxidative stress and inflammation. Under our experimental conditions, Wistar rats treated with a single dose of 40 mg STZ/kg bw underwent a marked hyperglycemia (395–416 mg/dL).

The administration of HEYL (100 mg/kg/d) in diabetic animals reduced serum glucose. These results are in agreement with Aybar et al. (2001) [23] and Genta et al. (2010) [24] studies, in which different extracts preparations and doses of Yacon, administered orally, reduced glycemia in STZ-induced diabetic rats, although Raga et al. (2010) [25] demonstrated that a dose of 100 mg/kg bw of Yacon tea presents more potential activity on glycemic control. The rate of blood glucose reduction in the present study occurred in synergy with the normally functioning pancreatic cells. The DM group without treatment has the lowest concentrations of insulin in plasma. The HEYL promoted a slight increase of insulin concentrations (Figure 1(c)), even without significant difference when compared to untreated DM group, and HOMA-BETA (Figure 1(d)), suggesting regeneration of functional *β*-cells.

It is well known that hyperglycemia is the major cause of diabetic complications. Oxidative stress is one of the potential mechanisms by which hyperglycemia can result in diabetic complications [26]. Improvement of glycemic control that achieves near-normoglycemia can decrease the development and progression of its complications [25]. Regeneration or protection of pancreatic cells that were partially destroyed by STZ with increase of insulin concentrations in plasma and probably increase in the peripheral utilization of glucose could be factors that can explain the significant decrease of fasting blood glucose in the present study [27]. Additionally, some phytochemicals such as flavonoids and polyphenols have been found to be effective due to some other extrapancreatic mechanisms [28]. Further studies are in progress to establish the precise mechanism involved in the antihyperglycemic effect of HEYL.

It has been shown that the solvent used in the preparation of plant extracts can affect positively or negatively the biologically active principles of these plants [8]. Baroni et al. (2008) [29] showed that the hydroethanolic extract of Yacon leaves was the best extraction to promote reduction of glycemia in diabetic and nondiabetic animals. Also, the polyphenolics in Yacon leaves may regulate the free radical activity of STZ diabetes induction [10] and the pathogenesis of diabetes [30]. Plants rich in phenolic compounds have potential hypoglycemic effects [31, 32]. Ferulic acid, p-coumaric acid, caffeic acid, chlorogenic acid, protocatechuic acid, and quercetin were the highest compounds found in the extract. Jung et al. (2011) [33] found that low doses of onion peel hydroethanolic extract ameliorate hyperglycemia and insulin resistance in high-fat diet/STZ-induced diabetic rats in 8 weeks of treatment. Additionally, Pereira Braga et al. (2013) [34] described that isolated quercetin promotes glucose regulation and decrease of lipid peroxidation in diabetic animals. Caffeic and chlorogenic acids are known for their antioxidant and free radical scavenging properties [35]. Recently, caffeic acid in particular has been associated with reduced blood glucose [36]. Baroni et al. (2016) [37] showed that the phytochemical analysis of the hydroethanolic extract of Yacon identified the presence of phenolic compounds such as caffeic acid, ferulic acid, gallic acid, and chlorogenic acid, corroborating with the present study. The phytochemical profile may explain the antioxidant and antihyperglycemic activities noted in our study.

**Figure 3:**
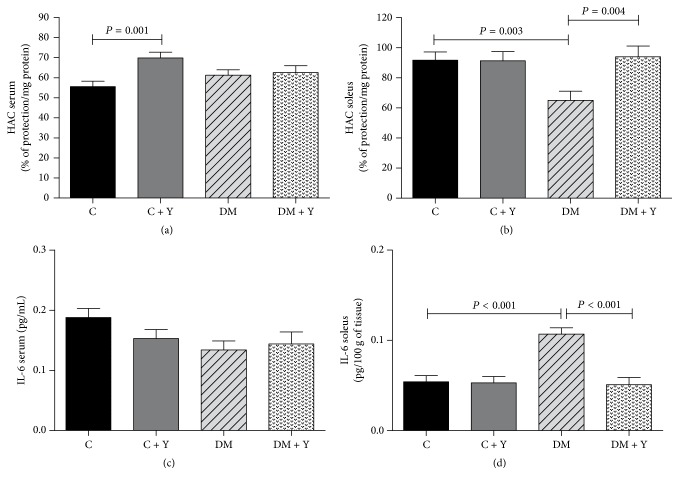
(a) Serum hydrophilic antioxidant capacity (HAC); (b) soleus HAC; (c) serum interleukin-6 (IL-6); (d) soleus IL-6 of the different experimental groups. C (control group): normal rats; C + Y: normal rats receiving HEYL; DM: diabetic rats; and DM + Y: diabetics rats receiving HEYL. The results are expressed as the mean ± SEM.

It is known that the pathogenesis of DM and its complications are associated with the overproduction of ROS and depletion of the endogenous antioxidant system, leading to oxidative stress [38]. It is also known that skeletal muscle is a primary tissue in the response to metabolic alteration inducing physiopathological stimulus. Several signaling pathways in striated muscle can be activated by an increase in ROS production [39]. HEYL increased the activity of catalase and GPx (Figure 3) in the soleus muscle is likely attributed to improvement in glucose oxidation or direct modulation of antioxidant enzymes. Some reports suggest that oxidative stress is a key player to diabetic complications, which may be associated with alterations in the metabolism [40, 41]. In addition, it has been reported that STZ induces severe oxidative stress in diabetic animals caused by the peroxidation of polyunsaturated fatty acids, leading to the formation of MDA as by-products of lipid peroxidation [42]. Excessive lipid peroxidation can readily attack the polyunsaturated fatty acids of the lipid membrane, which in turn can disrupt the structure of biological membranes and produce toxic metabolites such as malondialdehyde [43]. MDA is often used as a marker of oxidative damage [44, 45]. In summary, excess ROS overwhelm antioxidant defenses, leading to oxidative stress.

No significant alterations were found in the plasma of diabetic animals for IL-6 (Figure 2(c)) when compared with controls. Although we did not observe changes in oxidative stress and inflammation markers when they were systemically evaluated after Yacon treatment, the leaves efficiently reduced metabolic markers, such as hyperglycemia, and oxidative/inflammation stress in soleus muscle. In addition, we observed that HAC decreased, while MDA and IL-6 increased in the soleus of diabetic animals (Figure 3), showing the oxidative stress and inflammation in this disorder.

The antioxidant activities of various vegetables, fruits, and plants are mainly attributed to their content of phenolic compounds [46]. The radical scavenging activity of polyphenols depends on the molecular structure and the substitution pattern of the hydroxyl groups, the availability of phenolic hydrogens, and the possibility of stabilization of the resulting phenoxyl radicals via hydrogen donation or by expanded electron delocalization [47]. This radical scavenging ability of extracts could be related to the nature of phenolics, thus contributing to their election transfer/hydrogen donating system.

In the present study, for the first time, the significant increase of the antioxidant status (HAC) and endogenous antioxidant activities (GPx and CAT) and decrease of markers of lipid peroxidation (MDA) and proinflammatory cytokine (IL-6) in soleus muscle in diabetic rats treated with HEYL suggest the antioxidant and anti-inflammatory activity of Yacon extract in this tissue. These results indicate that Yacon leaves have significant effects on scavenging free radicals, promoting decrease of oxidative stress under diabetic conditions.

Although the HEYL promoted several benefits on STZ-induced diabetic model, especially those regarding the glucose homeostasis and antioxidant activities, this study has limitations. The precise mechanisms by which HEYL promoted antihyperglycemic activity and increase of insulin concentrations have not been evaluated, even if they are hypothetically attributed to regeneration/preservation of pancreatic beta-cells. But further studies are in progress to investigate the precise mechanism/pathways involved.

STZ administration induces hyperglycemia and increases MDA and IL-6 in soleus muscle, toxic intermediates in the development of oxidative stress and inflammation in diabetes. Moreover, experimental diabetes decreases the capacity of antioxidant defenses in soleus muscle. In summary, these results demonstrate hyperglycemia-induced oxidative stress in skeletal muscle of diabetic rats. In conclusion, the hydroethanolic extract from* S. sonchifolius* leaves (HEYL) protects against hyperglycemia, oxidative stress, and inflammation in skeletal muscle and also promotes increase of serum insulin concentrations in STZ-induced diabetic model in rats. These findings provide information that can guide future studies aimed at finding therapeutic alternatives for diabetic complications.

## Figures and Tables

**Table 1:** Phenolic content of hydroethanolic extract of Yacon leaves.

RT (min)	Name	Peak area (*µ*C)	Concentration
15.29	Protocatechuic acid	6.98	10.11
25.83	Gentisic acid	8.78	7.64
33.13	Chlorogenic acid	2.30	8.17
35.97	Vanillic acid	3.08	1.34
37.65	Caffeic acid	66.10	27.56
42.25	Epicatechin	2.20	5.11
46.67	p-Coumaric acid	19.50	169.81
49.78	Ferulic acid	7.05	13.21
50.43	Sinapic acid	6.41	4.68
75.35	Quercetin	128.0	399.00

RT: retention time. Concentration is expressed in *µ*g/100 mg of HEYL.
